# ﻿A new species of the genus *Plotina* Lewis, 1896 (Coleoptera, Coccinellidae) from Guangdong, China

**DOI:** 10.3897/zookeys.1260.164418

**Published:** 2025-11-21

**Authors:** Fang Lin, Xingmin Wang, Kuan Li, Chaorong Li, Zhendong Huang, Yan Yi, Yuhua Lin

**Affiliations:** 1 Guangdong Ruyang Forest Farm (Nanling Mountain National Forest Park Management Office), Shaoguan, Guangdong 512727, China Guangdong Ruyang Forest Farm (Nanling Mountain National Forest Park Management Office) Guangdong China; 2 College of Plant Protection, South China Agricultural University, Guangzhou, 510640, China South China Agricultural University Guangzhou China; 3 Administration of Guangdong Nanling National Nature Reserve, Shaoguan, Guangdong 512727, China Administration of Guangdong Nanling National Nature Reserve Guangdong China

**Keywords:** Checklist, imaginal structures, lady beetle, male genitalia, Nanling, Plotinini, taxonomy

## Abstract

The genus *Plotina* Lewis, 1896 (Coleoptera, Coccinellidae, Plotinini) is distributed in the Oriental region. To date, eight species have been described, all of which are currently recorded from China. A new species, *Plotina nanlingensis* Lin & Wang, **sp. nov.** is described from the Nanling National Nature Reserve in China. Morphological details and genitalia of the new species are presented. A checklist of all known species in this genus is also provided.

## ﻿Introduction

The genus *Plotina* Lewis, 1896 (Coleoptera, Coccinellidae, Plotinini) was established by Lewis (1896) to accommodate a single species from Japan, *P. versicolor* Lewis, 1896, which is characterized by a dorsal tricolor pattern. Mader (1931) examined a syntype of *P. versicolor* and reassigned the species to the tribe Coelopterini. This placement was adopted by Korschefsky (1931) in his catalogue. Subsequently, Mader (1955a) described *P. muelleri* from Fujian, China, and Kovář (1995) added a third species, *P. quadrioculata*, from Thailand. Miyatake (1969) revised the genus *Plotina* and related genera (*Sphaeroplotina* Miyatake, 1969, *Haemoplotina* Miyatake, 1969, *Palaeoneis* Crotch, 1874, *Paraplotina* Miyatake, 1969 and *Buprestodera* Sicard, 1911), suggesting that they exhibit a close phylogenetic affinity based on similarities in genital structures and many external structural characters. Later, Miyatake (1994) proposed the tribe Plotinini for the genus *Plotina* and the aforementioned related genera. Wang et al. (2011)described four additional new species of *Plotina*: *P. octomaculata*, *P. menghaiensis*, *P. daweishanensis* and *P. signatella*.

In this study, we report a new species of *Plotina* from Guangdong Province, China, providing a detailed description and illustrations. An updated checklist to the known species of *Plotina* is also presented.

## ﻿Material and methods

Examined specimens are deposited at the Department of Entomology, South China Agriculture University (**SCAU**), Guangzhou, China. All materials were preserved in 85% ethanol. External morphology was observed using a dissecting stereoscope (Zeiss Stemi 2000–CS). The following measurements were taken with an ocular micrometer: length from apical margin of clypeus to apex of elytra (**TL**); width across both elytra at widest part (**TW**); height at highest elytral part (**TH**); head width at widest part including eyes (**HW**); pronotal length at longest part (**PL**); pronotal width at widest part (**PW**); elytral length at longest part (**EL**); and elytral width across both elytra at widest part (**EW**). Male and female genitalia were dissected, cleared in a 10% solution of NaOH by boiling for several minutes, and examined with an Olympus BX51 compound microscope.

Photographs were imaged with a digital camera (Qimagin 5.0 RTV and Coolsnap–Pro*cf* & CRI Micro*Color) mounted on the dissecting microscope. Image capture was performed with the Image-Pro Plus 5.1 software, and photos were processed and arranged into plates using Adobe Photoshop CS 8.0. Morphological terminology follows Ślipiński (2007).

## ﻿Taxonomy

### ﻿Genus Plotina

Taxon classificationAnimaliaColeopteraCoccinellidae

Lewis, 1896

CE6549B3-9808-5834-873D-C1D8E6718AD6

Plotina Lewis, 1896: 35. Type species (original designation): Plotina versicolor Lewis, 1896.
Plotina : Mader 1927: 22; 1931: 200; 1934: 294; 1955b: 822; Korschefsky 1931: 213; Pope 1962: 632; Miyatake 1969: 198; Sasaji 1971: 65; Pang and Mao 1979: 24; Hoàng 1982: 106; Miyatake 1994: 281; Kovář 1995: 58.

#### Distribution.

China, Japan, Thailand, Vietnam.

### ﻿ Plotina nanlingensis

Taxon classificationAnimaliaColeopteraCoccinellidae

Lin & Wang sp. nov.

6026557E-1286-5B57-A1DA-269D0C32EA6B

https://zoobank.org/6D7D9547-2F7D-413C-AF77-38683F087F41

Fig. 1a–h

#### Diagnosis.

Species closely resembles *P. muelleri* (Fig. 2a–h) in general morphology. The newly described species is separated from the latter based on the following features: *P. nanlingensis* has a markedly larger body, lacks two prominent rounded white spots on the pronotum, and a large, elongate black spot on the middle elytron. Penis guide of *P. nanlingensis* in ventral view is robust, arrow-shaped, with a concave base. In lateral view, the penis guide is subparallel along basal 2/3, then rapidly tapers to a point at the apex.

#### Description.

Measurements: TL: 5.37–5.93 mm, TW: 4.24–4.69 mm, TH: 2.34–2.89 mm, TL/TW: 1.26–1.27; PL/PW: 0.30–0.32; EL/EW: 1.05–1.06.

**Figure 1.**
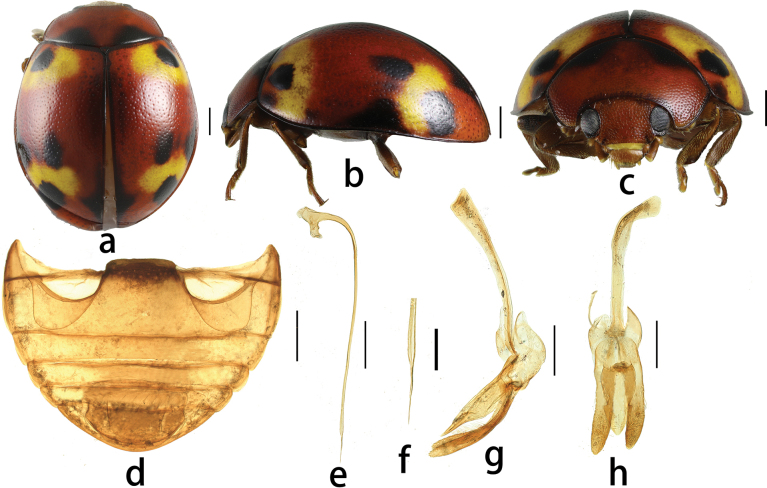
*Plotina nanlingensis* sp. nov. **a.** Dorsal view; **b.** Lateral view; **c.** Frontal view; **d.** Abdomen; **e–h.** Male genitalia: **e.** Penis, lateral; **f.** Apex of penis; **g.** Tegmen, lateral view; **h.** Tegmen, ventral view Scale bars: 1.0 mm (**a–c**); 0.2 mm (**d–h**).

Head reddish-brown, eyes dark brown (Fig. 1c). Pronotum dark reddish-brown, anterior angles yellow. Scutellum typically black. Ground color of elytra dark reddish-brown, and each elytron with 2 large yellow maculae and 7 black markings as shown in Fig. 1a.

**Figure 2.**
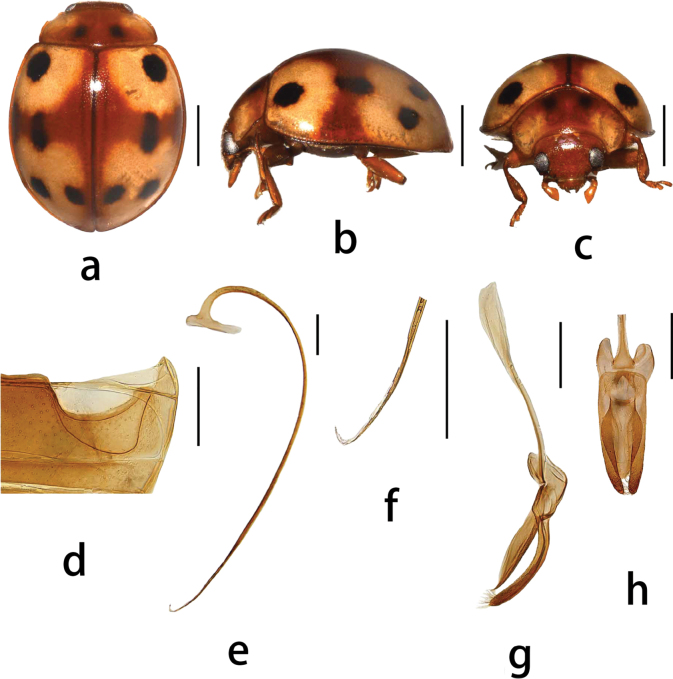
*Plotina muelleri* Mader **a.** Dorsal view; **b.** Lateral view; **c.** Frontal view; **d.** Abdomen; **e–h.** Male genitalia: **e.** Penis, lateral; **f.** Apex of penis; **g.** Tegmen, lateral view; **h.** Tegmen, ventral view. Adapted from Wang et al. (2011). Scale bars: 1.0 mm (**a–c**); 0.3 mm (**e–h**).

Body broadly oval, moderately convex. Head comparatively small, width 0.39 × elytral width (HW/EW = 2.57); punctures on frons moderately large, separated by 1.0–1.5 × their diameter, white setae in punctures; eyes small, broadly oval, widest interocular distance 0.54 × width of head. Pronotum 0.68 × elytral width (PW/EW = 1: 1.47). Pronotal punctures distinct, in two sizes, separated by 0.5–1.5 × their diameter. Punctures on elytra slightly larger than those on head, separated by 0.5–1.0 × their diameter. Punctures on metaventrite fine, 2.0–4.0 × their diameter, short setae in punctures. Abdominal postcoxal lines complete, almost touching posterior margin of ventrite 1 (Fig. 1d).

Male genitalia (Fig. 1e–h): Penis long, slender, penis capsule with both arms well developed; apex simple, pointed, and straight. Penis guide in lateral view, subparallel at basal 2/3, rapidly tapering to apex, apex pointed. Parameres slender, subparallel at basal 2/3, gradually weakly thickening, sparsely setose at apex, longer than penis guide. Penis guide in inner view, arrow-shaped with concave base, apex rounded, widest at basal third.

#### Specimens examined.

***Holotype*.** • 1 male, CHINA, Guangdong, Nanling National Nature Reserve, Shaoguan, 800 m, [24°55'42"N, 113°1'27"E], malaise trap, 28.ix.2022–7.i.2023, Huang Z.D. leg. (SCAU). ***Paratypes*.** • 7 males, same data as holotype (SCAU).

#### Distribution.

China (Guangdong).

#### Etymology.

The new species is named after its type locality, Nanling National Nature Reserve.

##### ﻿Checklist of the genus *Plotina*

***Plotina muelleri* Mader, 1955**

*Plotina mülleri* Mader, 1955a: 73; 1955b: 1026.

*Plotina muelleri*: Miyatake 1969: 202; Pang and Mao 1979: 24; Kovář 1995: 61; Wang et al. 2011: 57.

**Distribution.** China (Fujian, Guangdong, Guizhou, Hainan, Hunan, Sichuan); Vietnam.

***Plotina octomaculata* Wang et al., 2011**

*Plotina octomaculata*Wang et al., 2011: 57.

**Distribution.** China (Tibet).

***Plotina menghaiensis* Wang et al., 2011**

*Plotina menghaiensis*Wang et al., 2011: 57.

**Distribution.** China (Yunnan).

***Plotina versicolor* Lewis, 1896**

*Plotina versicolor* Lewis, 1896: 35; Mader 1931: 200; 1955b: 822; Nakane 1963: 209; Miyatake 1969: 200; Sasaji 1971: 66; Wang et al. 2011: 57.

**Distribution.** China (Anhui), Japan.

***Plotina daweishanensis* Wang et al., 2011**

*Plotina daweishanensis*Wang et al., 2011: 57.

**Distribution.** China (Yunnan).

***Plotina signatella* Wang et al., 2011**

*Plotina signatella*Wang et al., 2011: 57.

**Distribution.** China (Guangdong, Hainan).

***Plotina quadrioculata* Kovár, 1995**

*Plotina quadrioculata* Kovář, 1995: 58, Wang et al. 2011: 57.

**Distribution.** China (Yunnan), Thailand.

***Plotina nanlingensis* Lin & Wang, sp. nov.**

**Distribution.** China (Guangdong).

## Supplementary Material

XML Treatment for Plotina

XML Treatment for Plotina nanlingensis

## ﻿Additional information

### Use of AI

No use of AI was reported.

### Author ORCIDs

Kuan Li https://orcid.org/0009-0001-5963-9429

### Data availability

All of the data that support the findings of this study are available in the main text.
